# Computer-Aided Design to Improve the Thermal Stability of *Rhizomucor miehei* Lipase

**DOI:** 10.3390/foods13244023

**Published:** 2024-12-12

**Authors:** Rong Teng, Jin Zhang, Zhui Tu, Qinghua He, Yanping Li

**Affiliations:** 1 State Key Laboratory of Food Science and Resources, Nanchang University, Nanchang 330047, China; 407900220108@email.ncu.edu.cn (R.T.); zj980318@126.com (J.Z.); tuzhui@ncu.edu.cn (Z.T.); heqinghua@ncu.edu.cn (Q.H.); 2 Jiangxi-OAI Joint Research Institute, Nanchang University, Nanchang 330047, China; 3 International Institute of Food Innovation, Nanchang University, Nanchang 330200, China; 4 Jiangxi Provincial Key Laboratory of Agrofood Safety and Quality, Nanchang University, Nanchang 330047, China

**Keywords:** *Rhizomucor miehei* lipase, computer-aided design, rational design, thermal stability, molecular dynamics simulations

## Abstract

Lipase, a green biocatalyst, finds extensive application in the food sector. Enhancing the thermal stability of lipase presents both challenges and opportunities within the food industry. This research employed multiple rounds of cross-screening using tools like FoldX and I-Mutant 3.0 to strategically design mutations for *Rhizomucor miehei* lipase (RML), resulting in eight unique single-point mutation designs. E230I, N120M, and N264M have been confirmed experimentally to be potential combination mutation candidates. The resulting triple mutant N120M/E230I/N264M showed a higher thermal stability, with an optimum temperature of 55 °C, 10 °C higher than that of the wild-type RML. The half-life was extended from 46 to 462 min at 50 °C. Furthermore, the catalytic activity of N120M/E230I/N264M on camphor tree seed oil increased by 140% to 600 U/mg, which aids in the production of novel structured lipids. Using molecular docking and molecular dynamics simulations, we analyzed the molecular mechanism of enhanced thermal stability. This study validated the efficacy and dependability of computer-aided design to generate heat-resistant RML mutants and indicated that RML N120M/E230I/N264M lipase can be used as an effective biocatalyst for fat processing in the food industry.

## 1. Introduction

**Figure 1:**
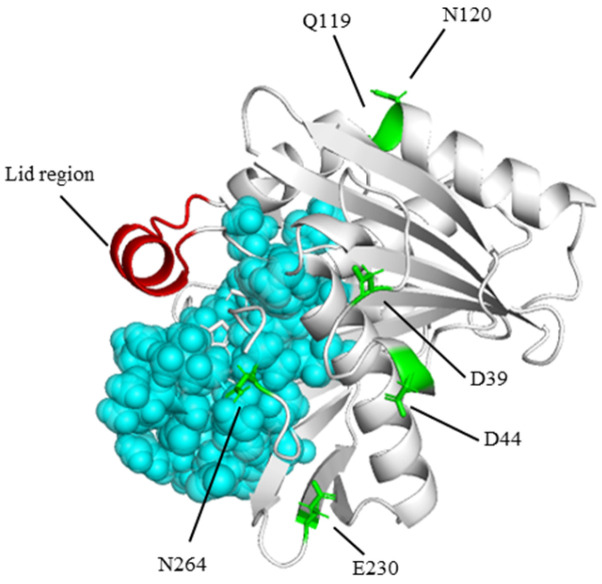
RML homology modeling diagram: the structure in red represents the lid, mutation sites are represented as green sticks, and residues within 5 Å of the catalytic triplet are represented as cyan spheres.

Lipases (EC3.1.1.3), also known as triacylglycerol hydrolase, are versatile enzymes that catalyze a range of reactions, including hydrolysis, esterification, transesterification, alcoholysis, and ammonolysis [1,2,3]. They are extensively used in the food industry, e.g., for the reuse of waste cooking oil [4,5], the improvement of the quality of baked cakes [6], the improvement of the flavor of fermented products [7], and the bioremediation of restaurant wastewater [8]. *Rhizomucor miehei* lipase (RML), consisting of 269 amino acid residues, is a stable enzyme with a molecular mass of 31.6 kDa and an isoelectric point of 3.8 [9]; it is characterized as a typical α/β-type single-chain lipase, with nine beta-pleated sheets and six alpha-helices (Figure 1). The catalytic triad of the active site—serine-histidine-aspartic acid (Asp203-His257-Ser144)—is shielded by a lid structure composed of amino acid residues from position 84 to 92. Unfortunately, the low thermal stability of natural RML renders its production costly and prevents its application in the food industry. According to Bommarius et al. [10], thermal stability is a crucial factor in determining whether enzymes are suitable for large-scale production. Therefore, improving the thermal stability of RML is essential for fulfilling industrial requirements. The seeds of the camphor tree (CK) have an annual yield exceeding 1 million tons in China [11]. More than 50% of the oil is derived from the kernels of camphor trees [11,12]. Approximately 95% of the medium-chain fatty acids in camphor tree seed oil (CKO) are commercially available as substitutes for cocoa butter [13] and plastic fat [14,15]. RML is a high-activity environmentally friendly medium/long-chain lipase that has the potential to catalyze the hydrolysis of camphor seed oil. Enhancing its thermal stability could significantly reduce production costs.

In the past decades, a growing number of researchers have dedicated their efforts to elucidating the mechanisms underlying the thermal stability of proteins and exploring strategies to improve their thermal stability. The interactions of aromatic rings, the number of hydrogen bonds, the number of disulfide bonds, hydrophobic interactions, the prevalence of salt bridges, the count of ionic bonds, and the distribution of amino acid composition have been identified as significant factors that influence protein stability [16]. Hydrogen bonding is the major force that constitutes the secondary structure of proteins, and the average energy of intra-protein hydrogen bonding is estimated to be approximately 0.6 kcal/mol [17]. Enhancing current hydrogen bond networks and introducing new intra-protein and protein-solvent hydrogen bonds through single amino acid modification proved effective [18]. The formation of disulfide bonds enables the protein to rapidly fold into a stable three-dimensional structure, reducing the entropy of the protein in its unfolded state, thus maintaining its structural stability [19,20]. A disulfide bond was introduced between 248V and 251T of *Penicillium cyclopium* lipase, and its half-life at 35 °C was increased by 12.8 times [21]. In aqueous media, protein folding tends to sequester hydrophobic amino acids toward the interior, a phenomenon known as hydrophobic interactions. The hydrophobic interactions primarily influence the conformational entropy of proteins [22]. Salt bridges are electrostatic interactions between positively and negatively charged residues carried by proteins. Lee et al. [23] introduced a novel approach for crafting precise salt bridges. This method raised the melting temperature (T_m_) by 15.7 °C by designing salt bridges on β-glucosidase’s surface at moderate temperatures. Overall, the thermal stability of enzymes is not determined by a single factor but is influenced by the synergistic effect of multiple factors [24].

To improve protein thermal stability, a variety of approaches have been utilized, including protein engineering [25], immobilization [26], and chemical modification strategies [27]. In the field of protein engineering, enzymes with enhanced stability can accommodate a broader spectrum of beneficial mutations, thereby possessing greater evolutionary potential [28]. Sun et al. [29] combined error-prone PCR and site-directed saturation mutagenesis to perform molecular modification of polyphosphate-dependent mannose kinase (PPGMK). The ideal mutant H92K/E119R has an enzyme activity that is twice that of the wild type and a half-life that is 5.4 times longer at 50 °C. Recent developments in improving thermal stability now utilize computer-aided rational design. Li et al. [18] employed Rosetta ddg_monomer, FoldX, and I-Mutant to predict stable mutants of RML, and they employed Disulfide by Design 2 (DBD2), SSBOND, MODIP, and BridgeD to predict and design potential disulfide bonds. Their results indicated that the engineered RML exhibited an apparent melting temperature and catalytic efficiency of approximately 15 °C and 40% greater than the wild-type enzyme, respectively. By performing molecular dynamics (MD) simulations of *Yarrowia lipolytica*-derived lipase Lip2 at different temperatures, Zhang et al. [30] selected residues from the common fluctuation sites for mutation. The optimum temperature (42 °C) of mutant V213P was 5 °C higher than that of wild-type LIP2, and its half-life was about 70% longer than that of the wild type. Building on a profound understanding of the structure and function of the enzyme, computer-aided rational design focuses on the identification of specific regions or residues of the enzyme, greatly reducing the number of target mutants [31]. Various computational strategies have shown success in enhancing enzyme thermal stability and offer a more precise and efficient method than conventional methods [32]. Computational tools like MD simulation, B-FITTER, Rosetta Design, FoldX, and I-Mutant 3.0 facilitate the prediction of mutations that enhance stability.

In this study, we aimed to improve the thermal stability of RML by computer-aided design. The improved RML was used to hydrolyze camphor seed oil to enhance the renewable utilization of waste oil. Using the RML homology model, we calculated changes in Gibbs free energy (ΔG) using advanced software tools such as FoldX and I-Mutant 3.0. Six point mutations were selected from predicted candidates for thermal stability identification and combined with beneficial mutational sites to further optimize the thermal stability of the lipase. Molecular docking studies and molecular dynamics simulations were conducted to gain insight into the mechanisms that enhance thermal stability.

## 2. Materials and Methods

### 2.1. Genes, Plasmids, and Strains

The *rml* gene was sourced from the plasmid pCAMBIA1300-Rab6-EGFP-RML, which is conserved in our laboratory. *Escherichia coli* DH5α served as the cloning host, while *E. coli* BL21(DE3) facilitated RML expression. The *rml* gene was integrated into the pET30(a) expression vector via NcoI and XhoI restriction sites.

### 2.2. Main Materials

The primers used for PCR amplification in this study are shown in Table S1. PrimerStar^TM^ HS DNA polymerase, *Nco*I restriction enzyme, and *Xho*I restriction enzyme were purchased from Takara Biotechnology (Beijing, China); kanamycin (Kan) and isopropyl-β-D-thiogalactopyranoside (IPTG) were purchased from Sangon Biotech (Shanghai, China); a 1 mL HisTrap^TM^ HP column was purchased from GE Healthcare Technologies (Chicago, IL, USA); 4-Nitrophenyl palmitate (pNPP), 4-Nitrophenyl myristate (pNPM), 4-Nitrophenyl laurate (pNPL), 4-Nitrophenyl decanoate (pNPD), 4-Nitrophenyl octanoate (pNPO), and 4-Nitrophenyl butyrate (pNPB) were purchased from McLean Biochemical Technology (Shanghai, China). The other reagents have no special instructions and are all commercially available as analytically pure reagents.

### 2.3. Construction of RML Mutants

The RML mutants were constructed by the overlap extension method [33] using primers listed in Table S1. For example, for mutant N120M, the following method was used: (1) upstream PCR amplification of template pCAMBIA1300::EGFP::RML as template and 644 and 638 as primers; (2) downstream PCR amplification of the template using pCAMBIA1300::EGFP::RML as the template and 637 and 630 as primers; and (3) full-length amplification: the upstream and downstream amplification products were purified and used as a template. The full-length amplification generated from the third PCR and plasmid pET30(a) were digested by *Nco*I and *Xho*I restriction enzymes. Then, both digested products were ligated and then transformed into *E. coli* DH5α. The mutations were confirmed by DNA sequencing.

### 2.4. Expression and Purification of Wild-Type and Mutant Proteins

*E. coli* BL21 (DE3) strains harboring either the pET30a-RML plasmid or its mutants were inoculated onto solid LB agar plates supplemented with kanamycin (50 μg/mL). The inverted plates were incubated at 37 °C overnight to promote the growth of colonies. Subsequently, a single colony was cultivated in 5 mL of the LB medium (i.e., the seeds) with shaking at 220 rpm for 12 h at 37 °C. Briefly, 500 mL of TB medium was inoculated with 5 mL of the seeds, and the mixture was repeatedly incubated until the optical density at 600 nm (OD600) reached 0.6–0.8. At this point, protein expression was induced by adding IPTG to a final concentration of 0.1 mM, followed by incubation with shaking at 160 rpm for 10 h at 14 °C. The fermentation broth was centrifuged at 7500 rpm for 20 min at 6 °C; then, the cell pellet was resuspended in 50 mL of lysis buffer (50 mM Na_2_HPO_4_, 0.3 M NaCl, 20 mM imidazole, and pH 8.0), and the cells were disrupted via sonication on ice (cycles of 4 s on and 6 s off for a total of 7 min). The lysate was centrifuged at 7500 rpm for 20 min at 4 °C, and the resulting supernatant was collected. The crude protein was filtered through a 0.45 µm membrane and loaded onto a Ni2+ affinity chromatography, employing a gradient elution with increasing concentrations of imidazole (20, 50, 200, and 500 mM) to specifically elute the target protein. The target protein was analyzed by sodium dodecyl sulfate polyacrylamide gel electrophoresis (SDS-PAGE) using 12% separating gel.

### 2.5. Enzymatic Property Assays of the Wild Type and Mutants

Both the determined alkali titration [34] and p-nitrophenol method [35] were utilized to assess lipase activity. One unit of enzyme activity (U) represents the amount of enzyme that generates 1 μmol of fatty acid (or p-nitrophenol) per minute, and the enzymatic activity (EA) is defined as U per mg of protein. We reported average values obtained from three measurements, with experimental errors below 15%. The optimal temperature was determined by examining the hydrolytic activity over a temperature range of 30 to 60 °C. By calculating the residual activity of samples heated to each temperature for 30 min, we evaluated the thermostability of the mutants. The half-life (t_1/2_) refers to the duration needed for the remaining enzyme activity to decline to half of the initial enzyme activity. This occurs after diluting the protein to the proper concentration and incubating at 65 °C for varying time intervals. Similarly, we determined the optimal reaction pH and pH stability by determining enzyme activity after incubation in the pH range of 6–10 and in different pH buffers. We also explored substrate specificity with various p-nitrophenyl esters. Kinetic parameters of mutants were evaluated in their ideal catalysis environment, adhering to the Michaelis–Menten model.

### 2.6. Determination of Enzymatic Activity of Wild-Type and Mutant RML on Camphor Seed Oil

The enzymatic activity of RML and its mutants to camphor tree seed oil was determined through alkali titration [34]. The camphor tree seed oil was extracted from camphor tree seeds by hot pressing according to the method of Wang et al. [36]. Then, the oil was centrifuged at 4000 rpm for 15 min and stored at 4 °C in the dark. To create a homogenous milky-white emulsion, we mixed camphor tree seed oil with 2% polyvinyl alcoho (PVA) (*v*/*v* = 1:3) and homogenized the mixture under high pressure for ten minutes. RML and its mutant’s enzymatic activity on camphor tree seed oil was assessed at 50 °C and pH 8.

### 2.7. Computational Analysis

#### 2.7.1. Homology Modeling of RML

SWISS-MODEL was used for homology modeling of RML (PDB: 6QPR), and PROCHECK program was used for model evaluation with Ramachandran plots. The quality of the 3D model was validated using the ERRAT and PROCHECK tools in the SAVES v6.0 web server. ERRAT analyzes the overall quality of the 3D model, and PROCHECK verifies the stereochemical quality by generating a Ramachandran plot of protein residues. If residues are in the most favored regions (90%) in the Ramachandran plot, it is a qualified model and can be used for further intelligent library construction and thermal stability mechanism exploration.

#### 2.7.2. Computational Design of RML Mutants

Based on the three-dimensional structure and sequence information of RML, we utilized the PositionScan module from FoldX [37] to conduct hypothetical saturation mutations on 269 residues and obtained the ΔΔG of all mutants (ΔΔG = ΔG_Mut_ − ΔG_WT_). Mutants with ΔΔG < 0 kcal/mol were retained; in the output results, negative ΔΔG values indicated stable mutations, and positive values indicated unstable mutations. Then, I-Mutant 3.0 and MUpro [38] were applied to assist in verifying the results filtered by FoldX. Finally, the joint screening results were re-evaluated using mCSM [39], SDM [40], DUET [41], and Epri [42].

#### 2.7.3. Evolutionary Conservation Analysis

Important residue sites associated with RML activity, including the catalytic triad, Ca^2+^ binding site, lid, and oxygen hole, were eliminated from the mutant library. The evolutionary conservation analysis of RML was obtained through the ConSurf server [43], and sites with conservation levels exceeding 8 were eliminated. In addition, PyMOL v2.5.7 was employed to further identify amino acid residues within 5 Å of the catalytic triad, which were also excluded to reduce the impact of mutations on enzyme activity.

#### 2.7.4. Residue Interaction Network Analysis

We examined the interaction networks between wild-type and mutant enzymes utilizing the RING 3.0 program, focusing on hydrogen bonds, van der Waals forces, salt bridges, and other pertinent interactions.

#### 2.7.5. Molecular Docking

The homology model of WT RML and mut-RML was established using SWISS-MODEL. P-nitrophenyl laurate (pNPL) was retrieved from the PubChem database (PubChem CID: 74778). Next, pNPL was docked into the active pockets of RML and mut-RML using AutoDock Tools (version 1.5.6). Water molecules were eliminated during protein treatment processing, followed by the addition of all hydrogen atoms and the distribution of the entire Gasteiger charge. The ligand (pNPL) received the same treatment to guarantee appropriate torsional rotation during docking. In the simulated RML and mut-RML proteins, a 40 × 40 × 40 Å grid box along the xyz-dimensions with grid spacing of 1.0 Å was set in the AutoGrid module, which can completely wrap the protein. Subsequent docking was performed using semi-flexible docking, set to 10 docking times, calculated using AutoDock Vina, and the optimal conformation of the lowest energy value (kcal/mol) was selected for subsequent molecular dynamics simulations. For Vina programs, we used default parameters unless otherwise noted. Finally, the docking results were visualized by PyMOL and Discovery Studio to determine the amino acid positions and the corresponding hydrogen bond distances.

#### 2.7.6. Molecular Dynamics Simulations

The preliminary structure of the RML P-NPL complex emerged through molecular docking. We conducted molecular dynamics simulations using YASARA (version 19.12.14) with the standard macro “md_run.mrc”. We used the AMBER03 force field for the protein, GAFF232 for pNPL, and TIP3P for water. If no other instructions were available, the default parameters were used. The RML was centered within a simulation cell, which extended 5 Å around all atoms utilizing periodic boundary conditions; Na^+^ and Cl^−^ were added into the system to neutralize the charge. The simulation system was performed at temperatures of 298 K, 318 K, or 328 K and a pH of 7.4 for a duration of 20 ns. The simulation snapshots were captured every 100 ps, and the YASARA “md_analyze.mcr” macro was utilized for trajectory analysis. The binding free energy of the ligand to the protein was analyzed using the “md_analyzebindenergy.mcr” macro.

## 3. Results and Discussion

### 3.1. Computational Design of RML

We performed homology modeling of RML (PDB: 6QPR) using SWISS-MODEL (https://swissmodel.expasy.org/ [accessed 20 May 2023]) and assessed the structure quality of model with PROCHECK. A protein structure is considered reasonable if no less than 90% of its residues fall within the most favorable regions, as per the Ramachandran plot analysis [44]. The homology model of RML showed that 96.97% of residues were distributed in the optimal rational region (Figure 1 and Figure S1A). The results show that our protein model is of high quality and suitable for the further construction of intelligent libraries and the exploration of thermal stability mechanisms.

For the computational design of RML, applications such as FoldX, I-Mutant 3.0, MUpro, MCSM, SDM, DUET, and EPri were utilized. The initial screening employed the “PositionScan” feature in FoldX (https://foldxsuite.crg.eu/command/PositionScan [accessed 20 May 2023]) to hypothetically saturate the mutagenesis of 269 amino acid residues of RML, preserving ΔΔG < 0 kcal/mol (ΔΔG = ΔG_Mut_ − ΔG_WT_) [37], leading to a total of 1110 mutants (refer to Table S2). These mutants are classified as stable mutants. ΔG is the Gibbs free energy change of a protein from its unfolded state to its folded state. In FoldX, the output file showed that ΔΔG < 0 kcal mol indicates that the free energy of the mutant is smaller than that of the wild type, and therefore, the mutated protein is more stable [37].

The second screening utilized I-Mutant 3.0 and MUpro [21] software, with overlapping mutations identified as positive results, leading to a total of 39 mutants (see Table S3). Unlike FoldX, the calculated ΔΔG in I-Mutant 3.0 and Mupro was positive for stable mutations and negative for unstable mutations. The final round of screening reassessed the results of the combined screen using MUpro [38], MCSM [39], SDM [40], DUET [41], and EPri, resulting in eight single-point mutants (refer to Table 1). Machine learning-based methods have been designed to predict mutation-induced changes in the thermal stability of proteins; however, all of these methods have limitations [45]. Different software based on different calculation methods using multi-software and multi-round screening can provide the correct mutation site to a greater extent, reducing the workload [45]. Using three computational tools, Xu et al. [46] predicted 12 site mutations, 11 of which made the carbonyl reductase LsCRm4 more stable.

**Figure 2:**
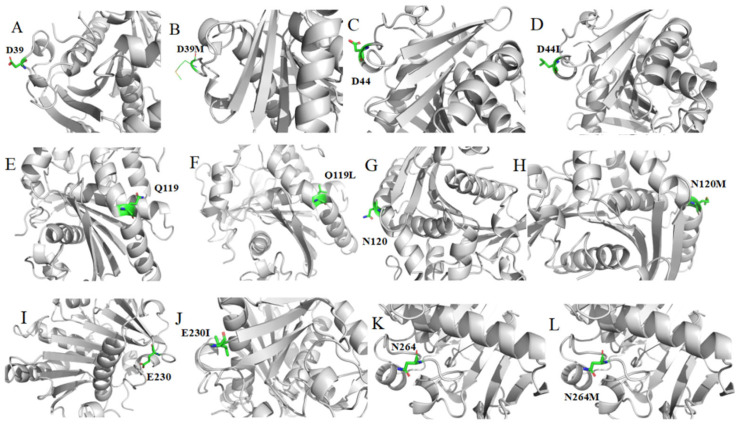
Structural position diagram of wild type RML and mutants. (**A**,**C**,**E**,**G**,**I**,**K**) the structure of wild type; (**B**,**D**,**F**,**H**,**J**,**L**) the structure of mutants D39M, D44L, Q119L, N120M, E230I, and N264M. Mutation sites are represented as green sticks.

In enzymes, mutations to catalytic residues are particularly unlikely to be tolerated as each of these residues is engaged in a very specific function during catalysis [47]. Utilizing ConSurf-DB, we evaluated the conservation scores of RML amino acid residues. We excluded residues with a conservation level exceeding eight from our mutation library (see Figure S2), which eliminated important residue sites associated with RML activity. These include the catalytic ternary, Ca^2+^ binding site, lid, and oxygen hole [43]. Moreover, we generated a single mutant for positions 120 and 230. Ultimately, we experimentally validated the mutations D39M, D44L, Q119L, N120M, E230I, and N264M (see Figure 2).

### 3.2. Expression and Purification of the Wild-Type RML and Its Mutants in E. coli

**Figure 3:**
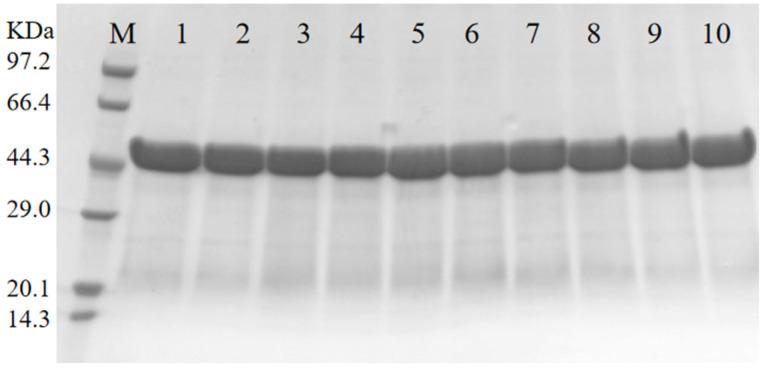
Expression and purification of wild-type RML and mutants. M: protein marker; 1: wild-type RML; 2: D39M; 3: D44L; 4: Q119L; 5: N120M; 6: E230I; 7: N264M; 8: N120M/E230I; 9: E230I/N264M; and 10: N120M/E230I/N264M.

The soluble expression of RML in BL21(DE3) is crucial for the thermal stability engineering of RML [48]. We took codon-optimized RML using the pET30(a) expression vector and increased the solubility of recombinant proteins using 0.1 mM IPTG cultured at 14 °C for 10 h [48]. Following the successful expression and purification of RML, we incorporated whole cells, samples of supernatant and granulosa cell post-rupture, and purified RML before and after IPTG induction by SDS-PAGE analysis for identification (Figure 3 and Figure S3). The molecular weight of the target protein was observed to be 44.3 kDa on SDS-PAGE gel, aligning with theoretical expectations. The crushed cells contained a large number of soluble proteins as well as the target foreign proteins, which were eluted by imidazole gradient using a Ni^+^ affinity chromatography column because the N-terminal of the target protein carried a His-tag; thus, the purified target protein was obtained. Subsequently, we employed the alkali titration method to assess the hydrolytic activity of wild-type RML, resulting in 230 U/mg. However, when Li et al. [18] used *E. coli* Rosetta-gami2 (DE3) expressed RML, its enzymatic activity was only 62 U/mg at 45 °C, which was much lower than that of this study (230 U/mg). The RML activity obtained from different expression strains will result in some differences. By employing high-yield strains of *Aspergillus oryzae* and optimizing fermentation parameters, researchers amplified RML activity to 320.0 U/mL [49]. The RML activity expressed by *Pichia pastoris* GS115 was 169.76 U/mL [6]. The lipase activity of the displayed RML reached 121.72 U/g in proteinase-A-deficient *P. pastoris* with a high-copy plasmid. The result was 46.7% higher than that of recombinant *P. pastoris* lacking a protease defect [50].

The single-point mutants were engineered using overlapping extension PCR with the primers listed in Table S1. After sequence verification of the mutant construct, the protein was expressed and purified. All the single-point mutants were successfully expressed in soluble form in *E. coli*, and the resulting protein bands matched the expected molecular weight. This indicates that the recombinant lipases were successfully secreted.

### 3.3. Enzymatic Characteristics of Single-Point Mutants

**Figure 4:**
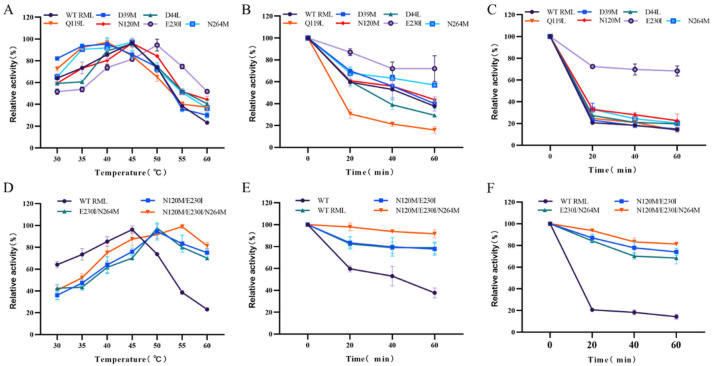
Characteristics of wild-type RML and mutants. (**A**) Optimum temperature of single-point mutants; (**B**,**C**) thermostability of single-point mutants at 50 °C (**B**) and 55 °C (**C**); (**D**) optimum temperature of combined mutants; and (**E**,**F**) thermostability of combined mutants at 50 °C (**E**) and 55 °C (**F**).

The optimal operating temperatures for the wild-type RML and its single-point mutants were determined by assessing enzyme activity within the range of 30–60 °C. The wild-type RML reached its peak activity at 45 °C (Figure 4A), which is the same as that observed in previous studies [9,18]. Among the mutants, the mutant E230I showed an elevated optimal temperature of 50 °C, while the optimal temperatures for mutants D44L, N120M, and N264M did not vary. Conversely, mutants D39M and Q119L displayed optimal temperatures that were lower than the wild type. Thermal stability was assessed by measuring the residual activity following incubation at 50–55 °C for 20, 40, and 60 min. At 50 °C, the relative enzyme activity of both the wild-type RML and its mutants decreased with prolonged incubation. Notably, mutants E230I, N264M, and N120M maintained over 50% activity after 60 min at 50 °C, indicating enhanced thermal stability (Figure 4B). In contrast, they exhibited a swift reduction in activity to approximately 30% after merely 20 min at 50 °C. After 20 min at 55 °C, E230I sustained around 70% of its activity, maintaining this level even at 60 min, which suggests improved thermal stability, while all other mutants show a drop to about 30% (Figure 4C). Overall, 50% of the mutants exhibited enhanced thermal stability, indicating that computer-aided design point mutations could be a promising approach to improving the thermal stability of enzymes.

By employing a range of computational design tools, such as FoldX, Li et al. [18] identified 36 single-point mutations, with 67% (24 out of 36) showing increased thermostability, and the E230I mutation displayed the most significant enhancement in thermostability. Our findings also suggest that E230I may be a potential hotspot residue. E230I improved the thermostability of lipase, which could be explained by the hydrophobic interaction effect [51]. At the E230 site, the hydrophilic glutamate was replaced by a hydrophobic L-isoleucine, leading to a more concentrated accumulation of hydrophobic regions and enhancing hydrophobic interactions in the mutant’s environment.

### 3.4. Characteristics of Combinatorial Mutants

To further enhance the enzyme’s properties, we strategically combined beneficial single-point mutations. Two two-point mutants, N120M/E230I and E230I/N264M, and a triple mutant, N120M/E230I/N264M, were generated by combining N120M or N264M and E230I, which had the highest residual activity. All combination mutants were expressed successfully in a soluble form in *E. coli*, showing molecular weights aligned with predicted values and purity levels surpassing 90% (see Figure 3).

Subsequently, the optimal temperature and thermal stability of these mutants were studied. We found that the optimal temperatures for the combination mutants N120M/E230I (50 °C), E230I/N264M (50 °C), and N120M/E230I/N264M (55 °C) showed a slight increase compared to the wild-type RML (45 °C) (Figure 4D). The catalytic efficiency of RML remained restricted to temperatures below 50 °C, thereby constraining its commercial viability at elevated temperatures [52]. The optimal temperature for N120M/E230I/N264M increased by 10 °C, providing specific guidelines for industrial production. The thermal stability of these enzymes was assessed by measuring their residual activity after 20, 40, and 60 min of incubation at temperatures of 50–55° C (Figure 4 and Figure S4). The activity of N120M/E230I/N264M remained virtually unchanged after 60 min at 50 °C, whereas N120M/E230I and E230I/N264M maintained approximately 80% of its activity (Figure 4E). Following a 60-min incubation at 55 °C, N120M/E230I preserved about 75% of relative enzymatic activity, while N120M/E230I/N264M sustained around 85% of relative activity, demonstrating superior thermal stability (Figure 4F).

The findings reveal that the combinatorial mutants N120M/E230I and E230I/N264M show greater thermal stability compared to their single-point variants, suggesting a synergistic effect. Furthermore, the triple mutant N120M/E230I/N264M demonstrated significantly enhanced thermal stability over both N120M/E230I and E230I/N264M, underscoring the beneficial impacts of combining mutations, which suggests the synergetic effect of the three amino acid residues of the triple mutant. The Computer-Assisted Recombination (CompassR) strategy guides the recombination of beneficial substitutions by analyzing the relative free energy of the fold and combining mutant residues in the active recombination (ΔG < +0.36 kcal/mol) group according to the ΔG values calculated by the FoldX method [53]. With an increase in the number of mutation combinations, the improvement of enzymes was gradually enhanced, including catalytic activity, stability, and resistance to harsh environments [53]. In this study, the ΔG values of the N120M, E230I, and N264M mutants were less than < +0.36 kcal/mol (Table S1, ΔG values were −0.35775, −1.59876, and −0.77627, respectively), which met the requirements of the active recombination group. The additive effect of mutations contributed to increased thermal stability, possibly due to mutant sites distant from the enzyme’s active site, and enhanced the hydrophobic interaction on the protein surface [51]. The hydrophilic L-Aspartic acid in the mutant sites N120M and N264M mutated into hydrophobic methionine, and the hydrophilic glutamate was replaced by a hydrophobic L-isoleucine, enhancing the hydrophobicity of the environment surrounding the mutant as well as the hydrophobic interaction effect [51]. Through these beneficial substitutions, enzyme variants with higher properties can be obtained. Yet, the recombination of multiple advantageous substitutions does not automatically yield enhanced enzyme variants. For instance, the stability of *Geobacillus stearothermophilus* Lipt6 L184F/A187F/L360F in methanol is significantly lower than that of Lipt6 L184F/L360F [54].

**Figure 5:**
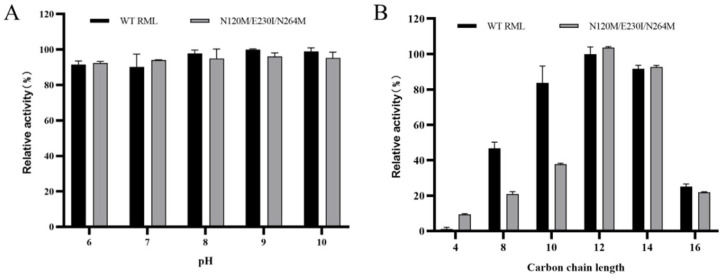
pH stability (**A**) and substrate specificity (**B**) of wild-type RML and mutant N120M/E230I/N264M.

The pH stability of wild-type RML and the mutant variant N120M/E230I/N264M was assessed over a pH range of 6.0 to 10.0. Both variants demonstrated considerable tolerance to alkaline conditions, retaining approximately 90% of their enzymatic activity at pH levels of 8.0 to 10.0 (see Figure 5A). Substrate specificity studies indicated that both the wild-type RML and the mutant N120M/E230I/N264M preferentially utilized p-nitrophenyl laurate (C12) as their primary substrate. The mutant N120M/E230I/N264M displayed higher specificity for both C12 and C14 (p-nitrophenyl myristate) compared to the wild type (refer to Figure 5B). In general, lipases are known to exhibit optimal activity against water-insoluble long-chain triacylglycerols (C8–C18) whereas esterases tend to hydrolyze water-soluble short-chain fatty acid esters (C8) [55]. Consequently, RML can be accurately classified as a bona fide lipase based on its substrate specificity.

As detailed in Table 2 and Figure S4, the half-life of the mutant N120M/E230I/N264M at 50 °C was significantly increased by a factor of 10.27, reaching 462 min, in contrast to the wild type, which had a half-life of 45 min. The immobilization of RML was achieved using the PIR-1 anchor system on the surface of *P. pastoris* cells. The optimal temperature for the biocatalyst RML-PIR1 was determined to be 45 °C, yielding an activity of 74.0 U/L. The half-life values at 40 °C and 45 °C were recorded as 3.49 h and 2.15 h, respectively [56]. The kinetic evaluation of the mutant was conducted under various optimal conditions, utilizing lauric acid p-nitrophenyl ester as the substrate. The higher K_m_ value indicates a reduced affinity between the enzymes and their substrates [57]. The K_m_ value was 92 μM for the wild-type RML and 31.75 μM for the mutant N120M/E230I/N264M. Compared to the wild-type RML, the mutant N120M/E230I/N264M exhibited a greater substrate affinity for p-nitrophenyl laurate, suggesting enhanced binding toward the substrate. This is similar to the result of Mohammadi et al. [58]. However, the affinity of RML to the substrate was related to the expressed strain; when RML was expressed in *P. pastoris*, the K_m_ reached 620 μM [9]. The enzyme activity of the mutant N120M/E230I/N264M was 320 U/mg, while that of the wild type was only 230 U/mg at the optimum temperature. This shows that the mutant N120M/E230I/N264M has strong hydrolysis capacity.

### 3.5. Enzymatic Activities of the Wild Type and Mutants to Camphor Tree Seed Oil

The camphor tree (*Cinnamomum camphora*) is commercially significant, with its seeds yielding over 40% camphor kernel oil (CKO) [14]. This oil is rich in medium-chain fatty acids, comprising more than 95% of its components, including 60.25% decanoic acid (C10:0) and 35.88% lauric acid (C12:0) [59]. Medium-chain and long-chain triacylglycerols (MLCTs) are novel structured lipids (SLs) that feature both medium-chain fatty acids (MCFA) and long-chain fatty acids (LCFAs) on the same glycerol backbone [60]. As a commercial edible oil, MLCTs can inhibit body fat accumulation and reduce cholesterol and blood triglyceride levels. In addition, excessive intake of MLCTs does not increase body fat accumulation [36,60]. CKO is rich in C12 and C10, which is a natural source of LCFAs and MCFAs [61].

To assess the enzyme activity of RML N120M/E230I/N264M to CKO, we extracted CKO from camphor tree seeds using a CKO emulsion as the substrate. The mutant variant N120M/E230I/N264M exhibited an enzyme activity of 600 U/mg, demonstrating a 140% increase compared to the wild-type RML, which had an enzyme activity of 427 U/mg. Ma et al. [13] synthesized a value-added cocoa butter substitute with fully hydrogenated palm oil and CKO by Lipozyme RM IM catalyzed esterification. Similarly, Tian et al. [62] also synthesized new functional trans-free lipids from CKO, pangasius bocourti stearin, and perilla seed oil via Lipozyme RM IM catalyzed esterification, which increased the added value of CKO, demonstrating the potential of lipase in green food processing. Because of the mutant’s higher specificity for C12 (Figure 5B) and higher enzyme activity to CKO, it is better for catalyzing hydrolysis, esterification, and transesterification of CKO via a lipase-catalyzed process to produce novel SLs. SLs differ from natural fats and oils in that the distribution and composition of their fatty acids (FAs) have been modified to meet the processing requirements of specialty foods [63]. Lipase-catalyzed transesterification has the advantages of mild reaction conditions, environmentally friendly characteristics, and low cost in the production of SLs [64].

### 3.6. Analysis of Residue Interaction Networks

Residue interaction networks (RINs) represent an alternative way to depict protein structures and have been extensively utilized to investigate mutation impacts, protein folding processes, inter-domain communication, and catalytic functions [65]. In this research, the online analysis tool RING predicted structural alterations in both wild-type and mutant RML, with the findings illustrated in Table 3.

The cationic-π interaction force and the disulfide bond count in the mutants correspond to those in wild-type Rhizopus lipase (wild-type RML). The newly introduced buried disulfide bond, F223C/G247C, can enhance the thermal stability of Rhizopus lipase (r27RCL) [66]. Additionally, new disulfide bonds S56C/N63C and V189C/D238C contribute 1.2 °C and 4.2 °C increases in Tm values, respectively, thus further bolstering the thermal stability of RML [18]. However, the incorporation of disulfide bonds into proteins does not invariably result in enhanced stability, making it challenging to predict suitable sites and their stabilizing effects [67]. Compared to the wild type, the number of hydrogen bonds at N120M/E230I/N264M increased by one, the number of salt bridges increased by one, and the number of van der Waals forces increased by nine (Table 3). Hydrogen bonding represents the primary force responsible for the formation of secondary structures in proteins. The enhancement of existing hydrogen bond networks through the introduction of single amino acid modifications has been demonstrated to be an effective approach [18]. In contrast to moderate-temperature proteins, heat-tolerant proteins display higher levels of charged amino acids. This abundance promotes the formation of surface salt bridges, thus creating a protective network on the protein surface. Such interactions enhance protein stability at elevated temperatures [68]. The N120M/E230I/N264M mutant introduced a new salt bridge, Glu16-Arg196, with the newly added salt bridge having a major contribution to the improvement of thermal stability. The van der Waals force is a weak intermolecular force that draws amino acid residues close to each other and stabilizes the three-dimensional structure of a protein [16]. The protein structure was more stable in the N120M/E230I/N264M mutant because it had nine more van der Waals forces. However, there was one less π–π stacking interaction than that observed in wild-type RML (Table 3). Aromatic interactions, including π stacking and π–π stacking within a distance smaller than 7 Å [16], which influence protein folding, often go unrecognized.

### 3.7. Analysis of Molecular Docking

**Figure 6:**
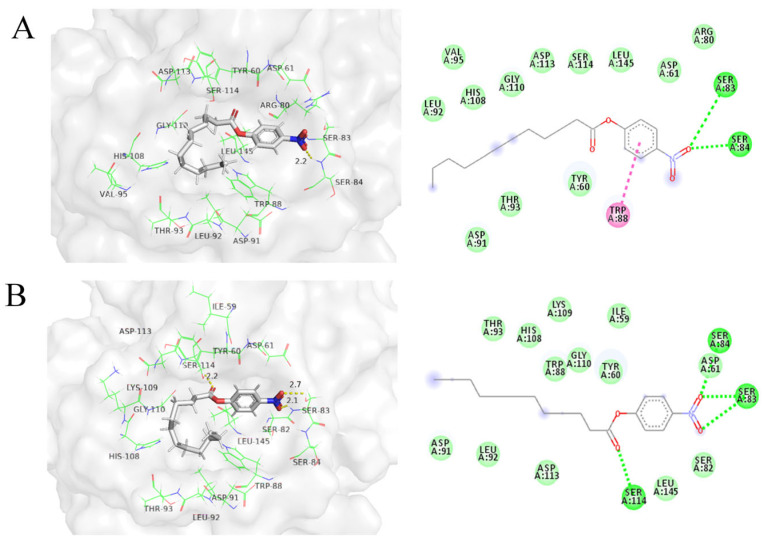
Analysis of molecular docking. Analysis of interactions between the wild type (**A**), N120M/E230I/N264M mutant (**B**), and pNPL. pNPL is shown as a gray sticks, the residues of 5 Å around pNPL shown as green line. Residues involved in Van der Waals forces are shown in light green, those involved in hydrogen bonding are dark green, and those involved in π–π stacking interaction are pink.

To deepen our understanding of the molecular mechanisms, we performed molecular docking experiments—docked wild-type RML alongside the three mutants N120M/E230I/N264M with nitrophenyl laurate (pNPL). The RML lid, a 15-amino acid helix spanning residues 82–96, reveals an active site resembling a shallow bowl when opened [69]. The lowest energy (kcal/mol) represents a simple estimate of better or stronger substrate affinity for enzymes in the enzyme–substrate conformation [70]. The binding energy of the triple mutant N120M/E230I/N264M was −5.6 kcal/mol, marginally lower than that of the wild-type RML (−5.5 kcal/mol). These results suggest that both the wild-type RML and mutant N120M/E230I/N264M can form a stable complex with pNPL. In the interaction between N120M/E230I/N264M and pNPL, two additional hydrogen bonds were formed, making the overall structure more stable (Figure 6B). The corresponding distances to two amino acid residues (Ser114 and Ser83) were 2.2 Å and 2.7 Å, respectively. The hydrogen bond interaction ensures the stable binding of pNPL to the lipase protein and affects the catalytic activity. Generally, weaker electrostatic interactions, such as NH–π, OH–π, CH–π, and π–π interactions, which influence protein folding, often go unrecognized [71].

The structural comparison of the wild-type RML with variant N120M/E230I/N264M is shown in Figure S5. Overall, the analysis revealed a high degree of similarity, with a root mean square deviation (RMSD) of only 0.023 Å. Three mutations, E230I, N120M, and N264M, on the surface of the enzyme and far away from the catalytic triad substitution altered the protein’s surface potential from a polar state to a relatively neutral state. Amino acids N (−3.5) and E (−3.5) of WT RML have low hydrophobic values (listed in parentheses after the residues). The hydrophobic values of the mutant were higher, with M (1.9) and I (4.5) [72]. The hydrophobic interaction between nonpolar residues, particularly Ile, can enhance protein stability [73]. This change increased the protein’s hydrophobicity, thereby enhancing its stability.

### 3.8. Analysis of Molecular Dynamics Simulations

**Figure 7:**
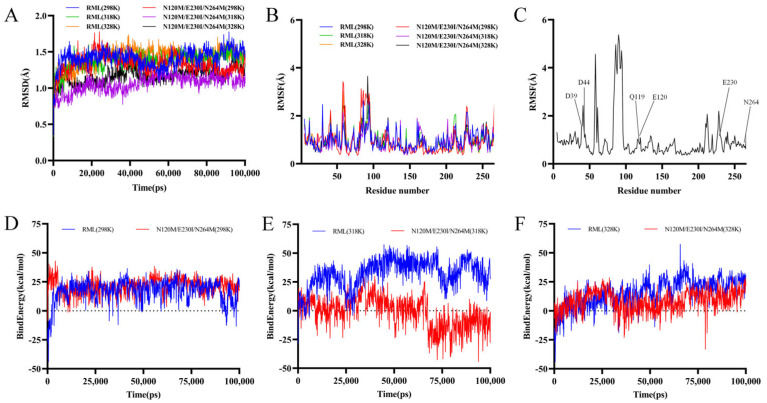
Analysis of Interactions between the wild type, N120M/E230I/N264M mutant, and pNPL. RMSD (**A**) and RMSF (**B**) values of the wild type and N120M/E230I/N264M mutant at 298 K, 313 K, and 343 K. (**C**) The positions of nineteen residues predicted by FoldX (shown by RMSF values). (**D**–**F**) The binding energy of the ligand pNPL to WT RML and mutant N120M/E230I/N264M during 10 ns of molecular dynamics at 298 K (**D**), 318 K (**E**), and 328 K (**F**).

A significant correlation exists between protein flexibility and optimal activity temperature [74,75]. To investigate the impact of mutations on the structural stability of E230IN120MN264M mutants, we conducted molecular dynamics simulations at 298 K, 318 K, and 328 K. Root Mean Square Deviation (RMSD) is a crucial parameter for evaluating thermal fluctuations in protein conformations. Lower RMSD values indicate greater thermal stability. The mean RMSD values for RML at 298 K, 313 K, and 343 K were 1.353 Å, 1.26 Å, and 1.223 Å, respectively (see Figure 7A). In contrast, the mean RMSD values for the N120M/E230I/N264M mutant at the same temperatures dropped to 0.098 Å, 0.329 Å, and 0.177 Å, respectively. This reduction in RMSD and the corresponding increase in the structural rigidity of N120M/E230I/N264M can account for its improved thermal stability.

Moreover, molecular dynamics simulations also provided insights into local flexibility within proteins. Increased Root Mean Square Fluctuation (RMSF) values for specific residues indicate higher conformational instability. For instance, the regions T57–T62 showed elevated RMSF values, signifying thermal instability (see Figure 7B). Therefore, mutations in these regions are likely to bolster the thermostability of lipases. By performing molecular dynamics (MD) simulations of *Yarrowia lipolytica*-derived lipase Lip2 at different temperatures, Zhang et al. [30] selected residues from the common fluctuation sites for mutation. The optimum temperature (42 °C) of mutant V213P was 5 °C higher than that of the wild-type LIP2, and its half-life was about 70% longer than that of the wild type. Similarly, regions S83–P96, part of the lid structure, displayed a sharp increase in RMSF values, suggesting considerable flexibility and reinforcing the critical role of the lid structure in lipases [76,77]. The RML lid is a 15-amino acid helix made from residues 82 to 96, with residues 83–84 and 91–95 acting as hinges [69]. The lid structure of lipase is also a hotspot mutation site for improving its catalytic activity. Zhang et al. [78] mutated the cover helix Asn87 and the hinge region Asp91, and the double mutant Asn87Ile/Asp91Val had a 130% higher hydrolytic activity (73 U/g) than WT.

Next, using 100 ns simulations at 298 K, 318 K, and 328 K, we determined the binding energy of the ligand pNPL to the lipase RML WT and RML N120M/E230I/N264M (Figure 7D). The binding energy of N120M/E230I/N264M was comparable to WT at 298 K. N120M/E230I/N264M had slightly lower binding energy than WT at 328 K and a significantly lower binding energy at 318 K. The reduced binding of RML N120M/E230I/N264M suggested that the enzyme had a higher relative activity and a better affinity for pNPL.

Finally, we contrasted residues predicted by FoldX with flexible regions identified by MD simulations. Utilizing FoldX, Mupro, and I-Mutant 3.0, we selected six positions for mutant residues (Table 1). Among these, four residues (D39, D44, N120, and E230) resided in regions with substantial fluctuations (Figure 7C), deemed thermally unstable. Notably, four single-point mutants (D39M, N120M, E230I, and N264M) demonstrated enhanced thermostability. Based on MD predictions, 75% (3/4) of these residues improved thermal stability, surpassing the 67% (4/6) predicted by FoldX. By using MD simulations, Zhang et al. chose two locations in the high-RMSF fluctuating region for mutation. The ideal temperature for mutant V213P is 42 °C, which is about 5.0 °C higher than WT LIP2, and its half-life at 50 °C is roughly 70% higher than WT LIP2 [30]. Using ΔΔG-based rational approaches, Wang et al. screened four heat-resistant variants, S142A, D217V, Q239F, and S250Y, in which D217V retained > 80% of the residual activity compared to WT [75].

All things considered, MD simulations clarified the molecular mechanisms underlying the mutants’ improved thermal stability, offering proof of their increased catalytic efficiency and improved substrate affinity. When predicting thermostable variants, merging FoldX with MD simulations proves to be more efficient, offering significant value for enhancing enzyme properties.

## 4. Conclusions

In this study, RML variants with improved thermal stability were predicted and designed using programs like FoldX, I-Mutant 3.0, MUpro, mCSM, SDM, DUET, and Epri. Initially, FoldX was used to analyze the saturation mutation of RML and assess the unfolding free energy change. Subsequent assessments using tools such as I-Mutant 3.0 pinpointed six single-point mutants that are likely to enhance thermal stability. Experimental validation showed that the single-point mutant E230I, along with N120M and N264M, greatly improved thermal stability. After obtaining the three-point mutant N120M/E230I/N264M, the activity was sustained over 80% after 60 min at 55 °C, exhibiting an optimal temperature of 10 °C greater than that of the wild type. Additionally, this mutant displayed significantly heightened substrate affinity and catalytic efficiency in comparison to the wild type. The K_m_ value of the wild-type RML was 92 μM, and that of the mutant N120M/E230I/N264M was 31.75 μM. Molecular docking and MD simulations further indicated that mutation could enhance the binding free energy between the substrate and the lipase. Furthermore, the catalytic activity of N120M/E230I/N264M for camphor seed oil increased by 140% to 600 U/mg, which was helpful for the synthesis of new structural lipids and the green and efficient conversion of waste oils. Furthermore, the mutant lipase’s remarkable heat resistance expands its potential application in the food industry and offers a valuable framework for enhancing thermal stability and other characteristics in enzyme preparations.

## Figures and Tables

**Table 1:** Mutants obtained by screening.

Mutation Site	Mcsm ΔΔG(kca/mol)	SDM ΔΔG(kca/mol)	DUET ΔΔG(kca/mo)	Epri ΔΔG(kca/mol)
D39M	0.343	0.29	0.456	−4.82
D44L	0.201	1.23	0.469	−0.85
Q119L	0.32	0.29	0.819	−2.06
N120L	0.066	1.52	0.463	−2.84
N120M	0.491	0.85	0.684	−4.86
E230L	0.324	0.69	0.755	−2.47
E230I	0.324	0.54	0.751	−2.83
N264M	0.531	0.55	0.745	−3.10

**Table 2:** t_1/2_ of wild-type *Rhizomucor miehei* lipase and mutants with selected beneficial mutations.

Mutants	t_1/2_ (min, 50 °C)	Folds
WT	45	1
D39M	46.21	1.03
D44L	33.65	0.75
Q119L	23.5	0.52
N120M	53.73	1.19
E230I	115.52	2.57
N264M	78.77	1.75
N120M/E230I	173.29	3.85
E230I/N264M	182.41	4.05
N120M/E230I/N264M	462	10.27

**Table 3:** Analysis of residue interaction networks for wild-type and mutant RML.

	HB ^a^	SB ^a^	π–π ^a^	DB ^a^	VW ^a^	π-C ^a^
WT	171	6	12	3	161	2
N120M/E230I/N264M	172	7	11	3	170	2

^a^: HB for hydrogen bonds, SB for salt bridges, π–π for π–π stacking, DB for disulfide bonds, VW for van der Waals forces, and π-C for π-cation interactions.

## Data Availability

The original contributions presented in the study are included in the article/Supplementary Materials, further inquiries can be directed to the corresponding author.

## Supplementary Materials

The following supporting information can be downloaded at: https://www.mdpi.com/article/10.3390/foods13244023/s1, Figure S1: Ramachandran plots of homologous models of wild-type RML and mutant N120M/E230I/N264M; Figure S2: Evolutionary conservation analysis of RML; Figure S3: SDS-PAGE analysis of wild-type and mutant protein purifications; Figure S4: Thermostability of wild-type RML and mutants; Figure S5: Structural alignment of wild-type RML (light grey) and variant N120M/E230I/N264M (in cyan); Table S1: Primers used in this study; Table S2: Electronic libraries of RML heat-stable mutants constructed using FoldX; Table S3: Electronic libraries of RML heat-stable mutants constructed using Mupro and I-Mutant 3.0.
