# Supplementary material for: Computer-Aided Design to Improve the Thermal Stability of Rhizomucor miehei Lipase
Source: Foods. 2024 Dec 12;13(24):4023. doi: 10.3390/foods13244023 (PMC11727178; doi:10.3390/foods13244023)
Supplement: Supplementary file 1 [file foods-13-04023-s001.zip › foods-3321203-supplementary.pdf]

Computer-aided Design to Improve the Thermal Stability of *Rhizomucor miehei* Lipase

Supplementary Materials

Figure S1: Ramachandran Plots of homologous models of wild-type RML and mutant N120M/E230I/N264M. We performed homology modeling of wild-type RML (A) and mutant N120M/E230I/N264M (B) by SWISS-MODEL, assessed the model structure using Ramachandran Plots, with 96.27% (wild-type RML) or 97.72% (N120M/E230I/N264M) of residues distributed in the optimal rational region, the structure of the protein is reasonable.

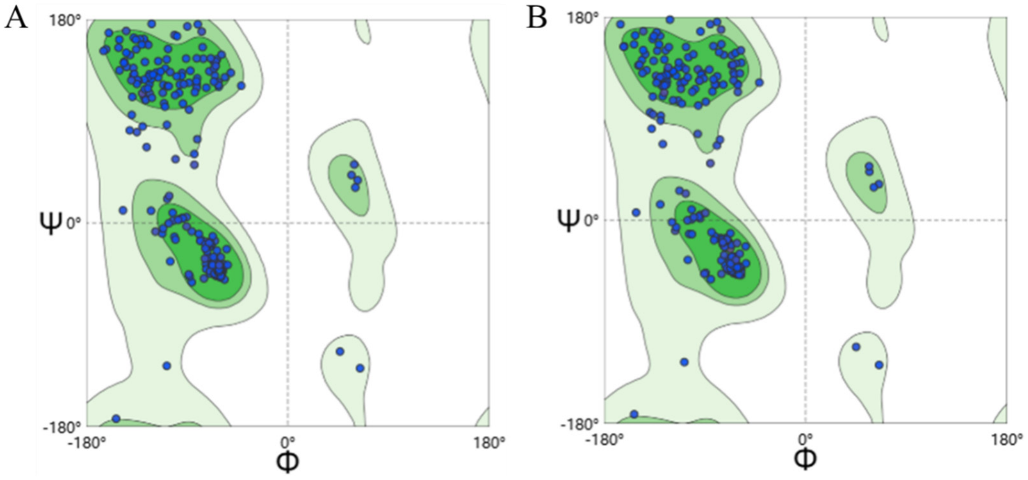

Figure S2: Evolutionary conservation analysis of RML. We use the Consurf-DB program to predict the evolutionary conservation of each RML amino acid residue. Color by conservative grade, the redder the color, the higher the conservative grade, and the less allowed the mutation.

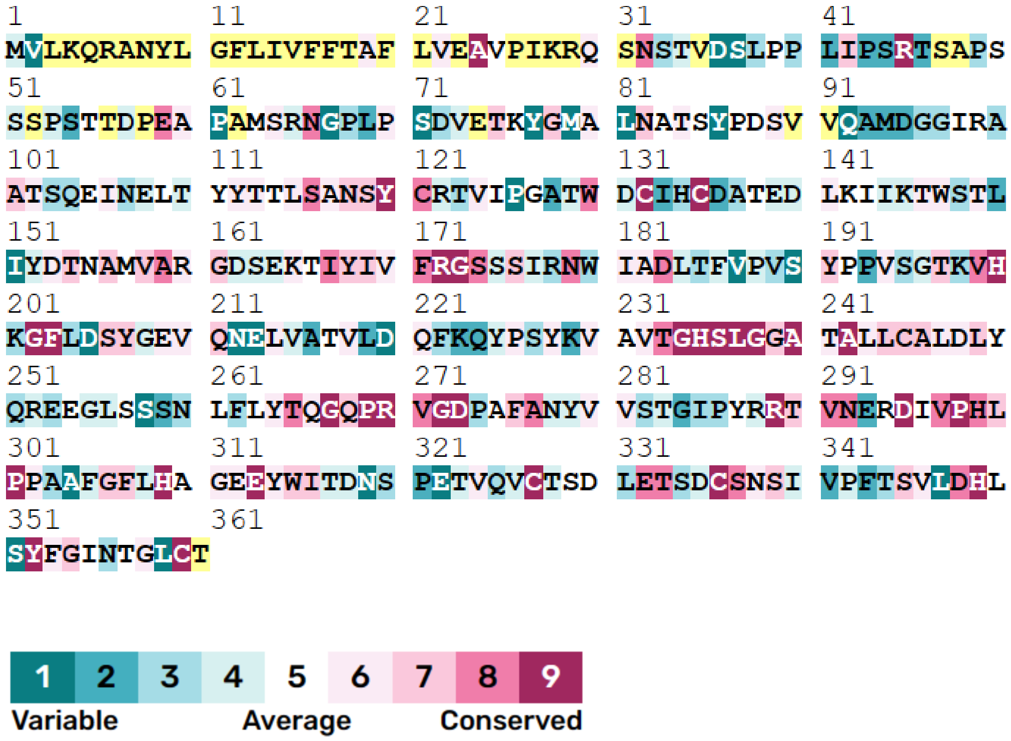

Figure S3: SDS-PAGE analysis of wild-type and mutant protein purifications. A, B, C, D, E, F, G, H, I, J: purification of RML- wild-type, RML-E230I, RML-N120M, RML-Q119L, RML-N264M, RML-D44L, RML-D39M, E230I /N120M, E230I/N264M; N120M/E230I/N264M; 1: protein Marker; 2-3: before and after bacteriophage induction; 4-5: broken bacteriophage supernatant and precipitation; 6: Flow-through; 7-9: 20 mM-500 mM imidazole elution.

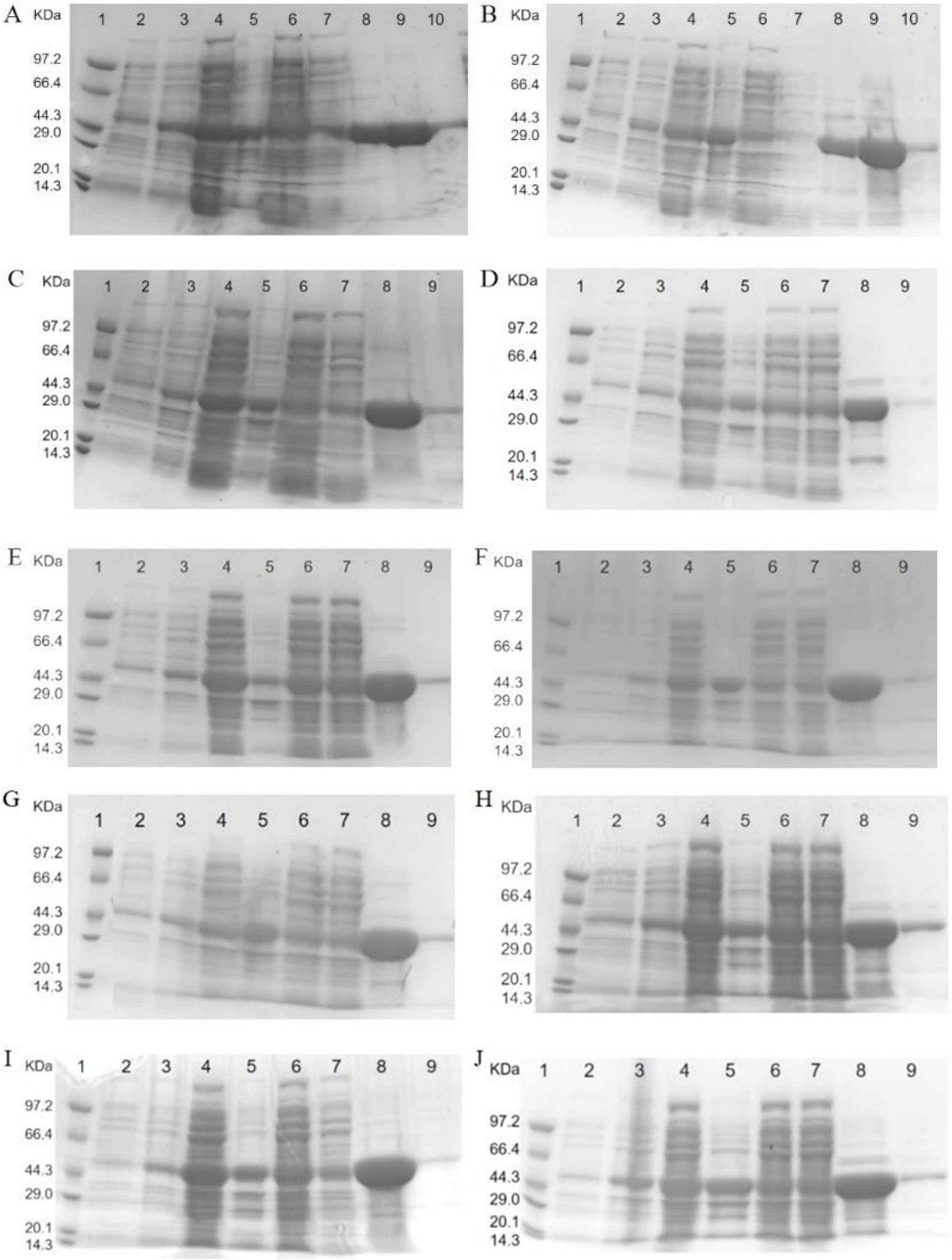

Figure S4: The thermostability of wild-type RML and mutants. A, B: the thermostability of single point mutants at 40 °C(A) and 45 °C(B); C: the thermostability of combined mutants at 45 °C; D: Half-life of wild type and mutant at 50°C.

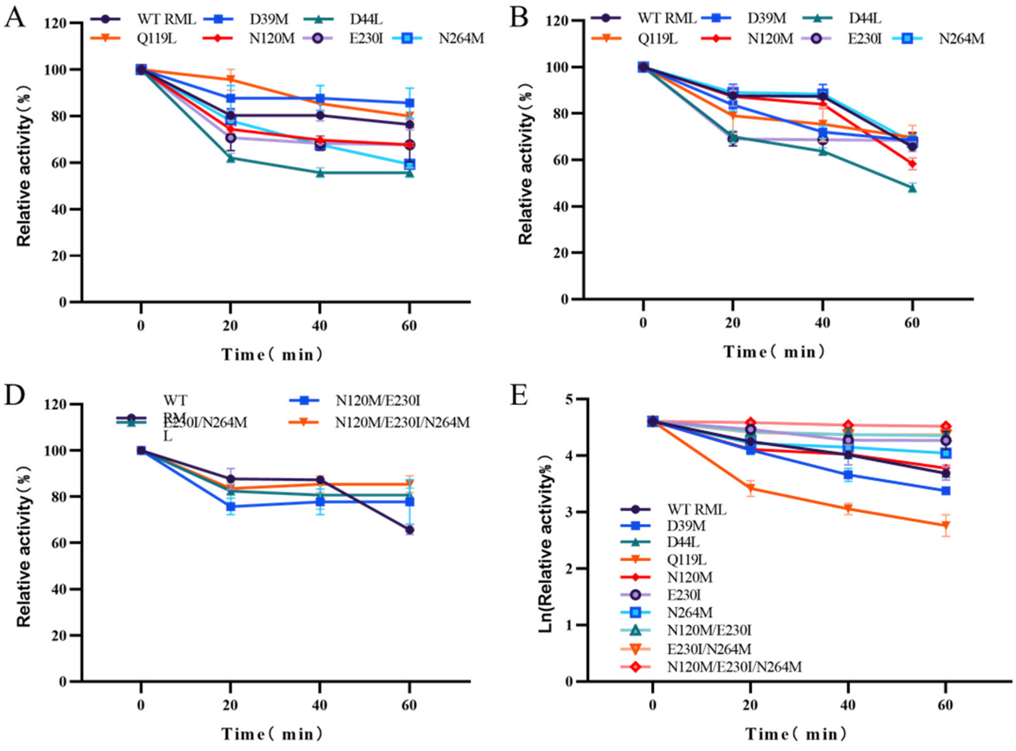

Figure S5: The structural alignment of wild-type RML (light grey) and variant N120M/E230I/N264M (cyan). The structure of RML WT was highly similar to that of the variant N120M/E230I/N264M, with an RMSD of 0.023 Å.

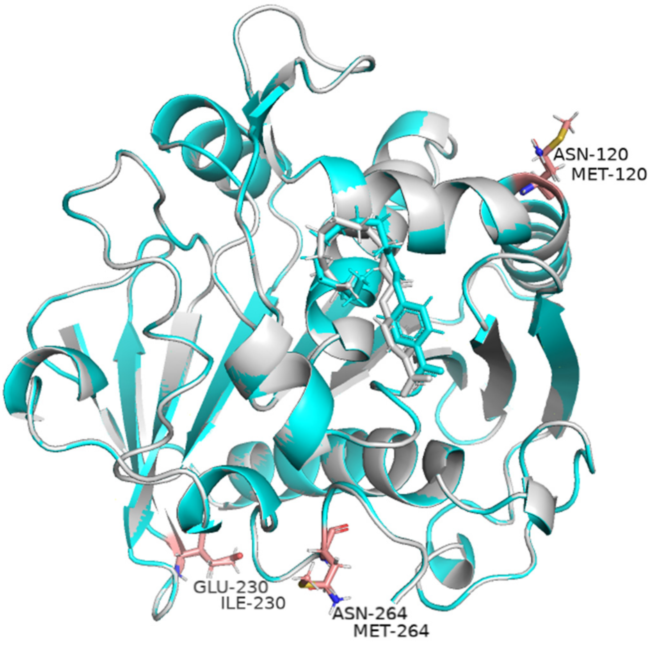

Table S1: Primers used in this study

Primer name	Primer sequences (5'-3')[a]
644-RML-HindIII-F	CCC*AAGCTT*GTGCCCATCAAGAGACAGTCCAA
630-RML-XhoI-R	CCG*CTCGAG*AGTGCAGAGGCCGGTGTTGATG
631-RML-D39M-F	CTACCTGG**ATG**TGTATTCACTGCGATGCAACGGAGGATC
632-RML-D39M-R	CAGTGAATACA**CAT**CCAGGTAGCTCCCGGAATGACAGTG
633-RML-D44L-F	TGCATCCACTGT**CTG**GCAACGGAGGATCTCAAGATTATCA
634-RML-D44L-R	CGTTGC**CAG**ACAGTGGATGCAGTCCCAGGTAG
635-RML-Q119L-F	GGAGGTG**CTG**AACGAGCTGGTGGCTACTGT
636-RML-Q119L-R	CCAGCTCGTT**CAG**CACCTCCCCGTAACTGTCCAG
637-RML-N120M-F	GGTGCAG**ATG**GAGCTGGTGGCTACTGTGCTGGATCA
638-RML-N120M-R	GCCACCAGCTC**CAT**CTGCACCTCCCCGTAACTG
639-RML-E230I-F	TGACAACAGCCCG**ATT**ACTGTACAGGTCTGCACCAGCGATCT
640-RML-E230I-R	GACCTGTACAGT**AAT**CGGGCTGTTGTCAGTAATCCAGTA
641-RML-N264M-R	CCG*CTCGAG*AGTGCAGAGGCCGGT**CAT**GATGCCGAAGTACGA GAG

[a] Italics and bold indicate restriction sites and the position of mutation, respectively.

Table S2: Electronic libraries of RML heat-stable mutants constructed using FoldX

Mutation site	ΔΔG (kca/mol)	Mutation site	ΔΔG (kca/mol)	Mutation site	ΔΔG (kca/mol)
A8L	-1.27804	A8V	-0.52789	A8I	-1.12735
A8P	-0.14960	A8R	-0.50181	A8T	-0.45005
A8S	-0.17661	A8C	-0.45541	A8M	-0.93441
A8K	-0.57124	A8E	-0.45403	A8Q	-0.82229
A8D	-0.18744	A8N	-0.07361	A8W	-0.93847
A8Y	-0.79001	A8F	-0.86395	T10G	-0.80707
T10A	-0.49619	T10S	-0.95318	T10E	-1.40222
T10Q	-0.28109	T10D	-3.02882	T10N	-1.52439
S11A	-0.38361	S11L	-0.24383	S11V	-0.13890
S11I	-0.78873	S11P	-1.91522	S11T	-0.02199
S11M	-1.05859	S11K	-0.09900	S11E	-1.48116
S11Q	-0.62204	S11D	-0.56408	S11W	-0.48583
S11Y	-0.01190	S11F	-0.20096	Q12P	-1.08557
Q12E	-1.20432	Q12D	-0.16363	I14L	-0.15489
N15A	-0.13738	N15L	-0.29849	N15R	-0.21875
N15I	-0.09354	N15S	-0.19103	N15M	-0.41655
N15K	-0.13196	N15E	-0.26473	N15Q	-0.38331
N15W	-0.62369	N15Y	-0.17668	N15F	-0.17601
E16L	-0.52708	E16M	-0.50235	L17M	-0.15041
T18A	-0.81989	T18L	-2.00316	T18V	-1.46904
T18I	-2.31733	T18R	-1.39799	T18S	-0.49303
T18C	-0.42122	T18M	-2.43404	T18K	-1.71915

Continue Table S2

T18E	-0.89629	T18Q	-1.25278	T18N	-0.04079
T18W	-2.11677	T18Y	-1.23944	T18F	-1.43063
Y19M	-0.65127	Y19F	-1.23191	Y20L	-0.14083
Y20F	-0.35806	T21L	-1.05225	T21V	-1.75966
T22A	-0.43440	T22L	-2.03103	T22V	-1.80221
T22I	-2.47548	T22R	-0.79774	T22C	-0.10208
T22M	-2.02644	T22K	-0.86115	T22Q	-0.26851
T22W	-1.82541	T22Y	-0.53228	T22F	-0.84470
S24A	-1.00310	N26A	-0.36332	N26L	-0.75627
N26V	-0.28560	N26T	-0.24026	N26S	-0.11382
S27A	-0.40052	R30P	-0.63560	T31A	-0.58817
T31L	-0.59226	T31P	-1.84376	T31R	-0.70481
T31S	-0.22134	T31M	-1.00255	T31K	-0.38920
T31E	-0.33575	T31Q	-0.71544	T31N	-0.69739
T31W	-0.45931	T31Y	-0.40531	T31F	-0.70785
T31H	-0.32768	A36G	-0.37623	A36R	-0.37385
A36S	-0.07122	A36C	-0.07989	A36M	-0.21957
A36K	-0.43230	A36Q	-0.23189	T37L	-0.02624
T37R	-0.31158	T37S	-0.13529	T37M	-0.17671
T37K	-0.51007	T37Q	-0.10078	T37F	-0.03102
T37H	-0.00190	D39R	-0.60222	D39M	-0.41030
D39K	-0.33183	D39Q	-0.33055	D39N	-0.00331
D39W	-0.08902	D39Y	-0.16744	D39F	-0.14300
I41A	-0.11189	I41P	-1.08986	I41W	-0.04896
H42L	-1.27690	H42I	-0.49611	H42R	-2.53995
H42M	-0.93459	H42K	-0.70338	H42Y	-0.89710
H42F	-1.20573	D44G	-0.33810	D44A	-0.56518
D44L	-0.39671	D44V	-0.22837	D44I	-0.33275
D44P	-0.86813	D44R	-0.60986	D44T	-0.30881
D44S	-0.35061	D44C	-0.16052	D44M	-0.36073
D44K	-0.58359	D44E	-0.28343	D44Q	-0.85258
D44N	-0.25901	D44W	-0.18228	D44Y	-0.58085
D44F	-0.77351	D44e	-0.06040	A45L	-1.22994
A45I	-0.35367	A45R	-1.96502	A45M	-1.28534
A45K	-0.37690	A45Q	-0.33697	A45W	-1.14429
A45F	-0.75041	T46L	-0.84279	T46V	-1.66978
T46R	-0.58372	T46M	-0.79818	E47G	-0.22643
E47A	-0.65781	E47L	-0.37642	E47I	-0.20342
E47R	-0.77516	E47S	-0.67712	E47C	-0.20224
E47M	-0.09628	E47K	-0.84458	E47Q	-0.54805
E47N	-0.43402	E47H	-0.63652	D48G	-0.51165
D48A	-0.45392	D48L	-0.71591	D48V	-0.10732
D48I	-0.54659	D48P	-0.34745	D48R	-0.90901

Continue Table S2

D48S	-0.54279	D48C	-0.52701	D48M	-0.55341
D48K	-0.84677	D48E	-0.10252	D48Q	-0.35692
D48N	-0.40966	D48W	-0.58332	D48Y	-1.76042
D48F	-1.09048	L49M	-1.03039	K50L	-0.14943
K50I	-0.15819	K50M	-0.25268	K50W	-0.08747
K50Y	-0.08956	K50F	-0.24504	I52Y	-0.82296
K53L	-0.38052	K53R	-0.25654	K53S	-0.16947
K53Y	-0.13092	K53F	-0.19057	T54V	-1.53439
T54I	-0.91168	T54C	-0.58157	T54M	-0.12409
S56V	-0.33861	S56R	-0.13945	S56C	-0.29874
S56M	-0.33807	S56K	-0.11927	S56Q	-0.28914
T57L	-0.08428	T57R	-1.16988	T57M	-1.02935
T57K	-0.68387	L58P	-0.35150	L58R	-0.02465
L58S	-0.12621	L58K	-0.14353	I59L	-0.59089
I59R	-1.06496	I59K	-1.02297	I59E	-0.24644
I59Q	-0.30054	I59W	-0.23762	I59Y	-0.43746
Y60F	-0.59425	D61G	-0.57890	D61S	-0.02963
D61Q	-0.06276	D61N	-0.70813	T62L	-0.55205
T62M	-0.52136	N63A	-0.14945	N63L	-0.72865
N63C	-0.14844	N63M	-0.57549	N63W	-1.38552
N63Y	-1.62850	N63F	-1.18962	A64T	-0.01627
A64S	-0.00880	V66I	-0.55744	A67V	-1.52657
A67I	-1.46968	A67M	-0.53111	R68I	-0.47457
G69A	-0.04097	G69L	-1.83532	G69N	-0.43346
G69Y	-1.28247	G69F	-0.38525	S71G	-0.04456
S71A	-0.28377	S71L	-0.20479	S71V	-0.03181
S71I	-0.13749	S71P	-1.14661	S71R	-0.70757
S71M	-0.38410	S71K	-0.48952	S71Q	-0.26648
S71W	-0.05981	S71Y	-0.13171	S71F	-0.13016
S71H	-0.05486	E72M	-0.09078	T74L	-1.25509
T74V	-1.95532	T74I	-2.11018	T74R	-0.26426
T74C	-1.21557	T74M	-1.12928	T74K	-0.72694
T74E	-1.01050	Y76F	-0.15879	V78L	-0.37036
V78I	-0.95949	V78M	-0.17859	R80A	-0.26156
R80V	-0.22561	R80T	-0.18704	R80C	-0.64041
R80M	-1.48195	R80K	-1.49654	S82A	-0.28877
S82L	-0.25567	S82P	-3.07508	S82R	-0.08653
S82C	-0.11494	S82M	-0.01101	S82K	-0.48759
S82E	-0.29667	S82Q	-0.07049	S82D	-0.51419
S83L	-0.17706	S83P	-0.41340	S83R	-0.18142
S83M	-0.39517	S83K	-0.22695	S83E	-0.05168
S83Q	-0.14256	S83D	-0.18226	S83N	-0.49302
S84E	-0.18753	S84D	-2.36735	S84N	-0.37083

Continue Table S2

I85E	-0.32936	R86L	-0.37099	R86I	-0.18520
R86P	-1.30770	R86M	-1.27282	R86K	-0.06629
R86E	-0.68186	R86Q	-0.08925	R86D	-0.13811
R86W	-0.80426	R86F	-0.20452	N87A	-0.48420
N87L	-0.47886	N87V	-0.12505	N87I	-0.59931
N87P	-2.07311	N87R	-0.47784	N87T	-0.39542
N87S	-0.38292	N87M	-0.59386	N87K	-0.37920
N87E	-1.01761	N87Q	-0.52458	N87D	-0.11423
N87W	-0.14274	N87Y	-0.15504	N87F	-0.17701
A90R	-0.30768	A90M	-0.91366	A90K	-0.34267
A90Q	-0.17677	A90N	-0.10937	A90W	-1.08229
A90Y	-0.38428	A90F	-0.48829	D91G	-0.71946
D91A	-0.26306	D91L	-0.77947	D91I	-0.44101
D91R	-0.46694	D91S	-0.21895	D91C	-0.55311
D91M	-0.79609	D91K	-0.68184	D91E	-0.28521
D91Q	-0.55874	D91N	-1.04730	D91W	-0.24295
D91Y	-0.75003	D91F	-0.84307	D91H	-0.90610
L92M	-0.75388	T93G	-0.36310	T93A	-0.42025
T93L	-0.73923	T93V	-0.45351	T93H	-0.45270
T93I	-0.32963	T93P	-0.67928	T93R	-0.95190
T93S	-0.28529	T93C	-0.45235	T93M	-1.37002
T93K	-1.15298	T93E	-0.56481	T93Q	-0.83394
T93D	-0.71807	T93N	-0.35542	T93W	-0.27317
T93Y	-0.49619	T93F	-0.56344	F94L	-0.00477
F94M	-0.11322	F94W	-0.03690	V95L	-0.65960
V95I	-0.22665	V95R	-0.57257	V95C	-0.35639
V95M	-0.88813	V95K	-0.77708	V95E	-0.26298
V95Q	-0.02941	V95D	-0.14365	V95N	-0.00093
V95W	-0.57443	V95Y	-1.11965	V95F	-1.24614
P96L	-0.21071	V97I	-0.56047	S98A	-0.17793
S98L	-0.80409	S98V	-0.23405	S98I	-0.79144
S98P	-1.74262	S98R	-0.36671	S98T	-0.06045
S98C	-0.10375	S98M	-0.45505	S98K	-0.71380
S98E	-0.82050	S98Q	-0.31243	S98D	-0.15698
S98N	-0.31068	S98W	-0.71336	S98Y	-0.88487
S98F	-0.97833	Y99F	-0.01749	V102L	-0.28147
V102I	-0.70560	V102M	-0.39940	S103A	-0.12147
S103V	-0.00170	S103P	-1.13806	S103R	-0.56840
S103T	-0.06113	S103K	-0.26377	S103E	-0.14911
S103Q	-0.00026	S103D	-0.14359	S103F	-0.00132
T105M	-0.41134	H108L	-1.66003	H108V	-0.96844
H108M	-1.31458	H108E	-3.52957	H108D	-4.77276
H108N	-1.21688	H108Y	-1.21621	H108F	-1.31945

Continue Table S2

K109L	-0.33408	K109M	-0.48153	G110A	-0.89934
G110L	-2.00257	G110V	-0.73373	G110I	-1.81551
G110P	-0.18689	G110R	-1.82477	G110T	-0.11733
G110S	-0.26946	G110C	-1.18107	G110M	-1.91081
G110K	-1.99931	G110E	-1.22687	G110Q	-0.96453
G110D	-0.40469	G110W	-2.07793	G110Y	-1.18928
G110F	-1.41403	L112M	-0.37234	L112W	-0.84571
D113A	-0.50488	D113L	-1.57968	D113R	-1.10586
D113S	-0.26945	D113M	-1.42430	D113K	-0.91906
D113E	-1.07188	D113Q	-0.73193	D113W	-0.56092
D113Y	-0.80940	D113F	-0.93212	S114A	-0.52302
S114L	-1.09401	S114V	-0.49065	S114I	-0.74909
S114R	-0.80475	S114C	-0.07856	S114M	-1.90343
S114K	-1.19173	S114N	-1.13483	S114W	-0.87424
S114Y	-0.61452	S114F	-1.10756	Y115M	-0.71558
Y115W	-1.44254	Y115F	-0.62841	G116A	-0.77242
G116L	-1.73771	G116I	-0.27212	G116R	-1.22675
G116S	-0.17692	G116C	-0.31238	G116M	-2.00948
G116K	-1.34872	G116E	-0.33954	G116Q	-0.83156
G116N	-0.76717	G116W	-1.52963	G116Y	-1.68201
G116F	-2.09948	G116o	-0.63843	E117A	-0.34169
E117L	-1.28331	E117V	-1.59726	E117I	-1.93728
E117R	-0.44208	E117T	-0.83808	E117C	-0.65132
E117M	-1.20441	E117K	-0.76970	E117Q	-0.59058
Q119A	-0.51852	Q119L	-0.66984	Q119V	-0.00073
Q119I	-0.73030	Q119R	-1.08290	Q119S	-0.77732
Q119M	-1.24395	Q119K	-0.90052	Q119E	-0.84732
Q119D	-2.12146	Q119Y	-0.06180	Q119F	-0.84283
N120A	-0.40207	N120L	-0.31824	N120I	-0.08916
N120P	-1.18913	N120R	-0.29909	N120T	-0.05518
N120S	-0.06624	N120M	-0.35775	N120K	-0.28636
N120E	-0.82470	N120Q	-0.79963	N120D	-1.02146
N120W	-0.22385	N120Y	-0.17358	N120F	-0.21801
E121M	-0.27228	V123L	-0.98890	V123I	-0.55647
V123M	-0.94472	V123E	-0.31114	A124L	-0.20393
A124R	-0.33408	A124M	-0.50162	A124E	-0.11755
A124Q	-0.20173	A124N	-0.09874	A124W	-0.04086
A124Y	-0.03731	A124F	-0.05895	T125L	-0.83011
T125V	-1.20017	T125I	-1.47877	T125M	-1.59764
T125K	-0.26706	T125Q	-0.34703	T125N	-0.18572
T125Y	-0.54578	T125F	-0.94680	L127M	-0.51683
D128A	-0.35609	D128L	-0.55896	D128I	-0.50459
D128R	-0.99498	D128S	-0.30586	D128M	-0.74637

Continue Table S2

D128K	-0.61145	D128E	-0.39834	D128Q	-0.61582
D128N	-0.43848	D128W	-0.62255	D128Y	-0.39864
D128F	-0.62892	D128H	-0.13833	Q129L	-1.91030
Q129V	-0.69946	Q129I	-1.93580	Q129R	-0.16673
Q129M	-0.98975	F130L	-0.89635	F130I	-0.59248
F130M	-0.15924	K131R	-0.42093	K131Q	-0.16250
K131Y	-0.18734	K131F	-0.22836	Q132L	-0.03509
Q132R	-0.00053	Q132K	-0.06406	Q132E	-0.17715
Q132N	-0.06532	Y133L	-0.01301	Y133R	-1.39566
Y133M	-0.11757	Y133K	-0.19707	Y133F	-0.75848
P134R	-0.80020	P134M	-0.06612	P134K	-0.78314
P134W	-0.18420	P134Y	-0.33827	S135P	-0.66104
S135R	-0.13199	S135C	-0.03406	S135K	-0.10490
S135W	-0.20431	S135Y	-0.09118	S135F	-0.12521
K137R	-0.50519	K137M	-0.48367	K137Q	-0.02129
V138L	-0.90610	V138I	-0.99917	A139V	-1.21288
A139I	-0.36855	A139T	-0.19425	A139C	-0.88865
A139M	-1.79382	A139K	-0.89612	V140L	-0.00861
T141V	-0.38791	H143A	-0.32655	H143L	-1.37695
H143V	-0.88982	H143I	-0.18196	H143T	-0.92356
H143S	-0.84300	H143C	-0.48718	H143M	-2.15619
H143N	-0.07354	S144G	-1.87874	S144C	-0.05370
L145R	-0.09094	L145M	-0.16459	L145D	-0.93111
T149L	-0.07492	T149V	-1.01398	T149I	-1.85592
L152M	-0.33139	C153A	-0.13893	C153L	-0.20403
C153E	-0.64686	C153Q	-1.53649	C153D	-0.18545
C153N	-0.35147	A154L	-0.84269	A154V	-0.50945
A154I	-0.52057	G156A	-1.55007	G156L	-2.55716
G156V	-1.02340	G156I	-1.80562	G156R	-1.75305
G156T	-0.32282	G156S	-2.38926	G156C	-0.85997
G156M	-3.04497	G156K	-1.11880	G156E	-0.91789
G156Q	-0.89269	G156D	-2.20086	G156N	-1.52859
G156W	-1.62639	G156Y	-2.35154	G156F	-2.67811
Y158L	-0.35055	Y158F	-0.29347	Q159L	-0.03382
E161L	-0.84212	E161I	-0.55534	E161M	-0.87826
E161K	-0.67703	E161Q	-0.55274	E162G	-0.13755
E162L	-0.00190	E162V	-0.08338	E162I	-0.10668
E162P	-1.40688	E162R	-0.46421	E162T	-0.10693
E162S	-0.02412	E162C	-0.11314	E162M	-0.17011
E162K	-0.26185	E162Q	-0.39013	E162N	-0.13468
E162W	-0.15701	E162Y	-0.25177	E162F	-0.30662
G163A	-0.19041	G163L	-0.85484	G163R	-1.75524
G163M	-0.93584	G163K	-0.75502	G163Q	-1.04120

Continue Table S2

G163N	-1.46500	G163Y	-0.78794	G163F	-0.66937
L164M	-0.58384	S165G	-0.03786	S165D	-0.98245
S165N	-0.08309	S166A	-0.71067	S166L	-0.61459
S166P	-2.54065	S166R	-1.38210	S166C	-0.06343
S166M	-0.56461	S166K	-1.18642	S166E	-0.58630
S166Q	-0.51683	S166D	-0.05461	S166N	-0.10600
S166W	-0.21101	S166Y	-0.29406	S166F	-0.32493
S167A	-0.30899	S167L	-0.57984	S167V	-0.00943
S167I	-0.22999	S167R	-0.39515	S167M	-0.63279
S167K	-0.61503	S167E	-0.27307	S167Q	-0.22556
S167D	-0.12517	S167Y	-0.01164	F170L	-0.03092
F170M	-0.05292	Y172F	-0.14425	Q174A	-0.07088
Q174L	-1.28506	Q174V	-1.70122	Q174T	-0.21400
Q174C	-0.59565	Q174M	-1.70911	Q174K	-1.57928
Q174D	-0.16966	Q174N	-0.23370	V179L	-1.38397
A183L	-0.24655	A183P	-1.92942	A183R	-0.27657
A183M	-0.26977	A183K	-0.02599	A183E	-0.11683
A183Q	-0.31682	A183N	-0.42563	N186L	-0.89072
N186I	-0.42076	N186R	-0.26897	N186M	-1.00945
N186K	-0.43248	N186E	-0.03513	N186Q	-0.17605
N186W	-0.78974	N186Y	-0.80556	N186F	-1.01149
Y187F	-0.13280	V188M	-0.20311	V189T	-0.09799
S190R	-0.29198	S190M	-0.03912	S190K	-0.21043
R196L	-1.49952	R196V	-0.07259	R196M	-1.71430
R196K	-0.94678	R196Q	-0.27837	R196W	-0.69634
R196Y	-0.82611	R196F	-1.14517	T198L	-0.30241
T198V	-1.72834	T198I	-2.97986	T198M	-2.16054
V199I	-0.16055	N200L	-0.88108	N200R	-0.98825
N200C	-0.12826	N200M	-1.44648	N200K	-1.17575
N200Y	-1.85274	N200F	-1.84029	N200H	-1.71178
E201G	-1.85130	E201A	-0.95380	E201L	-1.88467
E201R	-2.31217	E201S	-0.75141	E201C	-1.11227
E201M	-2.68765	E201K	-2.92068	E201Q	-1.43011
E201N	-1.92549	E201W	-1.68997	E201Y	-1.57266
E201F	-2.02813	V205L	-0.37720	V205I	-0.32312
V205M	-0.62224	L208M	-0.31783	A211L	-0.92753
A211I	-0.34874	A211P	-2.10260	A211R	-0.94572
A211M	-0.99626	A211K	-0.70909	A211E	-0.39378
A211Q	-0.84154	A211N	-0.38120	A211W	-0.52481
A211Y	-0.35708	A211F	-0.35528	A211H	-0.25419
A212L	-1.12477	A212V	-0.33733	A212I	-0.90784
A212P	-0.39068	A212R	-0.65005	A212C	-0.18880
A212M	-1.36466	A212K	-0.98832	A212E	-0.80512

Continue Table S2

A212Q	-0.45845	A212W	-0.57144	A212Y	-0.13327
A212F	-0.16559	F213L	-0.49177	F213M	-0.69302
F213W	-0.28035	L216K	-0.04710	L216F	-0.55311
A218P	-1.72451	E220A	-0.66577	E220L	-1.70415
E220V	-1.45268	E220I	-1.38473	E220P	-2.61020
E220R	-0.69676	E220T	-0.77542	E220S	-0.29872
E220C	-0.68173	E220M	-1.35057	E220K	-1.02438
E220Q	-0.46100	E220N	-0.66411	E220W	-0.19368
E220Y	-0.67513	E220F	-1.04583	E220H	-0.27453
E221L	-1.50010	E221I	-0.02987	E221P	-0.34219
E221M	-1.04148	T225L	-1.16827	T225V	-0.73917
T225I	-0.89639	T225R	-0.53831	T225C	-0.37700
T225M	-0.89871	T225K	-0.54318	T225Y	-0.58245
T225F	-0.65005	D226G	-0.13348	D226A	-0.35629
D226L	-0.22831	D226R	-0.78379	D226S	-0.92614
D226C	-0.16755	D226M	-0.30567	D226K	-0.18224
D226Y	-0.05730	D226F	-0.08739	D226H	-0.10249
N227L	-1.07434	N227I	-0.14993	N227P	-0.26544
N227R	-0.33600	N227M	-0.97957	N227K	-0.18347
N227F	-1.01824	N227H	-1.16420	S228L	-0.19261
S228R	-0.49068	S228C	-0.00179	S228M	-0.54575
S228K	-0.24807	S228Q	-0.25445	S228D	-0.02239
S228N	-0.33191	S228W	-0.05615	S228Y	-0.23821
S228F	-0.30876	P229G	-0.22317	E230L	-2.60772
E230V	-0.92715	E230I	-1.59876	E230P	-0.88066
E230R	-0.20918	E230T	-0.69053	E230C	-0.41773
E230M	-1.74083	E230K	-0.59049	E230D	-0.28204
E230Y	-0.24047	E230F	-0.29855	T231L	-0.48267
T231V	-0.56431	T231I	-0.86153	T231R	-1.66853
T231M	-0.46362	T231K	-0.92650	T231E	-0.30594
T231Q	-0.56636	T231D	-0.55543	T231N	-0.31065
T231W	-0.00108	T231Y	-0.52585	T231F	-0.58540
T231H	-0.25654	V232L	-0.72409	V232I	-0.92761
Q233L	-1.24567	Q233V	-0.06278	Q233I	-0.54059
Q233R	-0.61262	Q233M	-1.44779	Q233K	-0.99134
Q233W	-0.01466	Q233Y	-0.33665	Q233F	-0.44029
V234I	-1.07245	T236G	-0.70335	T236A	-0.93905
T236L	-1.79145	T236V	-0.85466	T236I	-1.25376
T236R	-1.00149	T236S	-1.14130	T236C	-1.39917
T236M	-2.11189	T236K	-1.08428	T236E	-0.92154
T236Q	-0.98478	T236D	-0.71638	T236N	-1.59361
T236W	-0.60860	T236Y	-0.82843	T236F	-0.78068
S237A	-0.19524	S237L	-0.40825	S237R	-0.92247

Continue Table S2

S237C	-0.04766	S237M	-0.61577	S237K	-0.64478
S237E	-0.03809	S237Q	-0.39887	S237D	-0.27456
S237Y	-0.27091	S237F	-0.25662	D238A	-0.37326
D238L	-1.85807	D238V	-0.57172	D238I	-0.45517
D238P	-1.15983	D238R	-3.36659	D238T	-0.10159
D238C	-0.10453	D238M	-2.17909	D238K	-0.96197
D238E	-1.43432	D238Q	-1.12933	D238W	-1.76945
D238Y	-2.21620	D238F	-2.27813	L239Y	-0.21225
T241L	-0.18675	T241V	-0.36490	T241R	-0.72392
S242G	-0.07340	S242L	-0.33467	S242P	-0.54216
S242R	-0.46502	S242M	-0.27195	S242K	-0.45915
S242Q	-0.08210	S242D	-0.04533	S242N	-0.17527
S242W	-0.25971	S242Y	-0.10571	S242F	-0.20462
D243L	-0.23605	D243M	-0.32033	S245A	-0.63446
S245V	-0.99666	S245P	-0.37965	S245C	-0.50114
S245M	-0.24053	N246W	-0.00396	S247R	-0.25112
S247M	-0.32929	S247K	-0.39692	V249L	-1.00572
V249I	-0.24718	V249P	-0.95965	V249R	-0.15030
V249M	-1.09341	V249E	-0.06818	V249W	-0.79675
V249Y	-0.64393	V249F	-1.15684	P250G	-0.27856
P250L	-0.19409	P250R	-0.17076	P250M	-0.18930
P250K	-0.18055	P250D	-0.14584	P250N	-0.14734
P250W	-1.50685	P250Y	-0.45377	P250F	-0.44836
F251R	-0.41203	F251M	-0.54831	F251Q	-0.10889
F251N	-0.43402	T252G	-0.28443	T252A	-0.94156
T252L	-3.04268	T252V	-1.73362	T252I	-1.80523
T252P	-0.74282	T252R	-2.08670	T252S	-1.13603
T252C	-0.82707	T252M	-2.99294	T252K	-1.96022
T252E	-0.64101	T252Q	-1.73283	T252D	-0.10488
T252N	-0.90100	T252W	-3.14184	T252Y	-2.14706
T252F	-2.51981	V254L	-0.92306	V254I	-0.51741
V254P	-0.10887	V254M	-0.86617	V254W	-0.35637
V254Y	-0.05233	V254F	-0.17894	L255P	-0.56645
L255M	-0.18124	D256E	-0.08794	H257L	-1.59828
L258M	-0.27383	L258F	-0.50469	S259R	-0.57456
S259Y	-0.02496	N264L	-0.43185	N264P	-1.44252
N264R	-0.26346	N264M	-0.77627	N264K	-0.42650
N264W	-0.75776	N264Y	-0.92435	N264F	-0.61516
T265A	-0.04043	T265L	-2.85264	T265V	-1.31981
T265C	-0.73591	T265M	-1.75415	T265D	-0.27530
T265N	-0.17225	L267I	-0.22857	L267R	-0.18620
L267M	-0.25496	L267E	-0.09322	L267D	-0.05968
L267Y	-0.06141	L267F	-0.19204	T269G	-0.97414

Continue Table S2

T269A	-0.30717	T269I	-0.10090	T269R	-0.15330
T269S	-0.08263	T269C	-0.20506	T269M	-0.36474
T269K	-0.24311	T269E	-0.27344	T269Q	-0.43010
T269W	-0.22101	T269Y	-0.43987	T269F	-0.09517

Table S3: Electronic libraries of RML heat-stable mutants constructed using Mupro, I-Mutant 3.0

Mutation site	Mupro ΔΔG(kca/mol)	I-Mutant ΔΔG(kca/mol)	Mutation site	Mupro ΔΔG(kca/mol)	I-Mutant ΔΔG(kca/mol)
S11L	0.55262216	0.65	N120L	0.22212365	0.42
S11V	0.1445936	0.65	N120I	0.21339003	0.64
S11I	0.37473791	0.44	N120M	0.06768243	0.41
S11M	0.21324499	0.50	N120F	0.02031586	0.50
S11F	0.20959848	0.62	D128I	0.043003442	0.27
D39M	0.061832405	0.07	K131Y	0.2510964	0.06
D44L	0.0095650322	0.15	K131F	0.4175347	0.45
E47I	0.002704478	0.24	T149I	0.42866347	0.16
D48L	0.054750662	0.40	Q159L	0.37426544	0.11
D48I	0.13298075	0.29	S166L	0.18321443	0.02
D48M	0.063504247	0.43	E220L	0.32232006	0.06
S103F	0.008396423	0.08	E220I	0.18976308	0.17
G116L	0.02690942	0.07	E220F	0.005100793	0.02
G116I	0.16178	0.01	S228L	0.034078908	0.02
Q119L	0.90163215	0.57	E230L	0.14130327	0.61
Q119V	0.5376988	0.60	E230I	0.058482256	0.58
Q119I	0.67757557	0.67	E230F	0.014757236	0.56
Q119M	0.53375849	0.19	N264L	0.16505071	0.51
Q119Y	0.32120133	0.28	N264M	0.084164573	0.62
Q119F	0.33299875	0.33			
